# Microbiome and Dental Changes in Horses Fed a High Soluble Carbohydrate Diet

**DOI:** 10.3390/ani15172547

**Published:** 2025-08-29

**Authors:** Milena Domingues Lacerenza, Júlia de Assis Arantes, Gustavo Morandini Reginato, Gabriela Luiza Fagundes Finardi, Pedro Henrique Marchi, Thiago Henrique Annibale Vendramini, Rodrigo Romero Corrêa, Pamela Aparecida Maldaner Pereira, Carlos Augusto Araújo Valadão, Renata Gebara Sampaio Dória

**Affiliations:** 1 Department of Veterinary Medicine, School of Animal Science and Food Engineering, University of Sao Paulo, Pirassununga 13635-900, SP, Brazil; mi_lacerenza@hotmail.com (M.D.L.); juxarantes@gmail.com (J.d.A.A.); gmorandinivet@gmail.com (G.M.R.); redoria@usp.br (R.G.S.D.); 2 Pet Nutrology Research Center (CEPEN Pet), Department of Animal Nutrition and Production, School of Veterinary Medicine and Animal Science, University of Sao Paulo, Pirassununga 13635-900, SP, Brazil; gabriela.finardi@usp.br (G.L.F.F.); pedro.henrique.marchi@usp.br (P.H.M.); thiago.vendramini@usp.br (T.H.A.V.); 3 Teaching and Research Support Center (CAEP), School of Veterinary Medicine and Animal Science, University of Sao Paulo, Pirassununga 13635-900, SP, Brazil; romero@usp.br; 4 Faculty of Agricultural and Veterinary Sciences, Sao Paulo State University, Jaboticabal 14884-900, SP, Brazil; pamela.maldaner@unesp.br

**Keywords:** equine dentistry, molecular microbiology, nutrition

## Abstract

This study investigated the oral microbiome of horses maintained on a high soluble carbohydrate diet based on sugarcane, in comparison to those fed a pasture-based diet composed of *Cynodon* spp., aiming to identify associations between dietary intake, the composition of oral microbial communities, and the occurrence of dental caries and diastemata. A total of 20 healthy horses, both male and female, with an average age of 9 ± 3 years and weight of 400 ± 100 kg, without a defined breed, were selected. They were divided into two groups: the High Soluble Carbohydrate Group (HSCCG), consisting of 10 horses fed sugarcane for at least 3 years, and the Low Soluble Carbohydrate Group (LSCCG), consisting of 10 horses fed pasture with *Cynodon* spp. Dental examinations were performed using an adapted oral endoscope to assess caries and diastemata, and saliva samples were collected for microbiota analysis. Statistical analyses included a Student’s *t*-test and Mann–Whitney test, with significance set at *p* < 0.05. Horses in the HSCCG had a higher presence of peripheral caries (*p* = 0.001), as well as differences in the degree (*p* = 0.010), class (*p* = 0.05), and presence of diastemata. Microbial diversity indices (Chao, inverse Simpson, and Shannon) showed no significant differences, but the relative abundance differed (*p* = 0.003). Linear Discriminant Analysis revealed distinct microbial profiles: Candidatus Saccharibacteria and Bacteroidetes were more abundant in the HSCCG, while Cyanobacteria_Chloroplast and Proteobacteria dominated in the LSCCG. Over 35 genera differed significantly between groups. The study concludes that diet plays a significant role in shaping oral microbiota and influencing the development of dental caries in horses.

## Simple Summary

This study aimed to evaluate the oral microbiome of horses fed a high soluble carbohydrate diet compared to those who were fed pasture grass (*Cynodon* spp.), relating findings to dental caries and diastemata by analyzing microbial profiles in conjunction with detailed dental examinations. The results showed that diet significantly influences oral microbiota and the development of dental caries in horses.

## 1. Introduction

Oral health is fundamental for the overall well-being of animals, and with the advancement of veterinary medicine and its specialties, equine dentistry has gained prominence in recent years. Dental changes in horses have multifactorial causes, with nutrition playing an important role among these factors. Over the years, with domestication and increased confinement, the diet of horses has undergone significant changes, becoming predominantly composed of concentrated feeds at the expense of forage [1]. In this context, the mechanics of chewing are directly affected, as is the chewing time, which is reduced, resulting in lower saliva production.

The predisposition to the development of dental caries and diastemas also occurs with the use of roughages high in soluble carbohydrates in the diet [2,3], since horses feed for up to 18 h per day, mainly on forage. If this forage contains simple carbohydrates, such as fructans, there is a high potential for maintaining a critical pH in the oral cavity for prolonged periods [4].

Another aggravating factor in the dental alterations currently observed in horses is the availability of roughage. Some of these feeds, frequently included in equine diets, are silages and forages with a high sugar content, such as sugarcane, which has been gaining more and more popularity as a roughage alternative for horses, mainly due to its low cost [5].

Dental caries is defined as the demineralization of inorganic calcified dental tissues, accompanied by the destruction of their organic component [3,6]. This process occurs due to the action of oral microorganisms, which possess an acidogenic capacity, converting fermentable carbohydrates into acids, resulting in damage to the teeth [7,8]. Moreover, caries can predispose the formation of diastemata, abnormal gaps between the teeth characterized by excessive or inappropriate separation between dental units [9], favoring food accumulation, fermentation, halitosis, and the development of periodontal diseases [3]. Such manifestations lead to dysbiosis, which, in turn, increases the predisposition to the proliferation of cariogenic microorganisms in the oral cavity [9].

In the normal oral cavity of animals, the natural microbiota is quite diverse, containing, among bacteria, species such as *Staphylococcus aureus*, *Staphylococcus mitis*, *Streptococcus oralis*, and *Streptococcus mutans* as the most commonly identified [10,11,12,13]. In horses, recent studies using next-generation genetic sequencing techniques have demonstrated differences in the oral microbiota profile of horses affected by periodontal disease in general and caries compared to healthy horses. It was found that the microorganisms most associated with periodontal disease were the *Prevotella* and *Veillonella* species, while those most associated with caries were the *Streptococcus*, *Veillonella*, and *Corynebacterium* species, whereas in healthy animals, the *Gemella* and *Actinobacillus* species were more commonly observed [8,14].

In humans, dental caries has been studied for many years, and the pathophysiology of the condition, as well as treatment and prevention, is well-established. However, in horses, these studies are scarce, especially regarding the oral microbiome, and there are no studies in the literature that demonstrate changes in the oral microbiota according to the type of food ingested, relating it to the presence of dental diseases. Therefore, this study aims to comparatively evaluate the incidence of peripheral caries, infundibular caries, and diastemata, and to determine the oral microbiological profile of horses fed roughage with high levels of soluble carbohydrates in comparison with those fed roughage from *Cynodon* spp. grass.

## 2. Materials and Methods

### 2.1. Ethical Statement

The research project was approved by the Animal Experimentation Ethics Committee of FZEA/USP and has the CEUA protocol number 1167131219.

### 2.2. Animals, Diets, and Experimental Design

Twenty animals, both males and females, healthy, with an approximate age of 9.0 ± 3.0 years and body weight ranging from 400 ± 100 kg, without a defined breed, were selected and divided into two experimental groups: the High Soluble Carbohydrate Group (HSCCG), consisting of 10 horses selected based on their forage source of sugarcane, regardless of the concentrate and mineral salt source, and the Low Soluble Carbohydrate Group (LSCCG), consisting of 10 horses selected for having a history of forage based on *Cynodon* spp. grass, regardless of the concentrate and mineral salt source.

The dietary history of the animals was obtained through interviews with the handlers regarding the forage source, concentrate, mineral salt, and their respective amounts provided within 24 h. All animals had the same feeding management for at least 3 years.

For oral cavity evaluation, the animals were restrained in a horse restraint chute. A dose of 0.05 mg/kg of acepromazine was administered intravenously (IV); after 30 min, the horses were given 20 µg/kg of detomidine IV, followed by a continuous infusion of 20 µg/kg/h, combined with 7 µg/kg/h of butorphanol IV.

With the horses sedated, the oral cavity was opened with the aid of an oral speculum, and samples were collected using sterile swabs from the occlusal surface of the maxillary teeth on their palatal, vestibular, and occlusal surfaces in both the LSCCG and HSCCG animals. The collected samples were placed in sterile tubes specifically for the swabs, sealed, and stored at a temperature of −80 °C.

### 2.3. Bromatological Analysis

Bromatological analyses were performed on both forage and concentrate samples used in the study. For forage sampling (including Purple Stargrass and Tifton 85), the sampled area within the paddock was marked, and between 15 and 20 subsamples were collected, covering the entire area. The upper portion of the vegetation, representing 50% of the pasture height, was cut using proper shears. These subsamples were then mixed to form a single, homogeneous sample. Approximately 1 kg of this composite sample was stored in plastic bags and frozen until bromatological analysis, as described by Genro and Orqis [15]. For sugarcane and Napier grass, samples were chopped before processing.

Regarding the concentrate samples, representative subsamples were collected from the feed used on each property. These subsamples were then combined into a single 1 kg composite sample, stored in plastic bags, and frozen until the analysis, also following Genro and Orqis [15].

All collected samples were dehydrated in a forced-air oven at 55 °C for 72 h until reaching a constant weight, followed by grinding in a Wiley mill (Thomas Scientific, Swedesboro, NJ, USA) with a 1 mm mesh screen. The contents of dry matter (DM), organic matter (OM), crude protein (CP), ash (MM), and ether extract (EE) were analyzed according to AOAC [16]. Starch content was determined using the enzymatic method described by Hendrix [17]. Soluble carbohydrate levels were assessed using the technique described by Silva and Queiroz [18], which involves spectrophotometric analysis after the formation of a blue–green complex produced by heating these compounds in a strongly acidic anthrone solution.

All analyses were conducted at the Multiuser Laboratory of Animal Nutrition and Bromatology of the Department of Nutrition and Animal Production, School of Veterinary Medicine and Animal Science, University of São Paulo. When the coefficient of variation among samples exceeded 5.0%, the analyses were repeated to ensure reliability.

### 2.4. Microbiota Analysis

DNA was extracted using the commercial QIAamp PowerFecal DNA Kit (Qiagen^®^, Germantown, MD, USA). PCR amplification was performed using the F515 and R806 primers for the V4 region of the 16S rRNA gene [19]. Both forward and reverse primers were designed to include a sequencing overlap region for Illumina (Illumina Inc., San Diego, CA, USA), so they could be recognized as primers with Illumina adapters containing more than 8 base pairs in the identification indexes [20].

The amplification was subjected to the following PCR conditions: 3 min at 95 °C for denaturation, followed by 35 cycles of 30 s at 95 °C for denaturation, 90 s at 55 °C for annealing, and 30 s at 72 °C for extension, followed by a final period of 5 min at 72 °C and maintained at 4 °C until purification.

The PCR products were purified in three steps, with the first two following the Illumina 16S Metagenomic Sequencing Library Preparation protocol, and the third step using a 2% agarose gel with the Zymoclean™ Gel DNA Recovery Kit Supplied with capped columns (Zymo Research^®^, Irvine, CA, USA) according to the manufacturer’s specifications. Next, the samples/libraries were quantified using the Qubit Assay Kit (Thermo Fisher Scientific, Waltham, MA, USA) according to the manufacturer’s recommendations. The purified and quantified libraries were mixed in an equimolar ratio and sequenced (2 × 150 bp) using the MiSeq^®^ Reagent Kit v2 300 cycles (Illumina Inc., San Diego, CA, USA) according to the manufacturer’s specifications, using the Illumina platform.

The bioinformatics analysis was performed using Mothur (version 1.31.2) following the MiSeq SOP, accessed in September 2021. Original fastq files were assembled into contigs, excluding sequences longer than 325 bp, those with base pair ambiguities, and those with homopolymers longer than 8 bp. The sequences were aligned using the SILVA 16S rRNA reference database. Chimeras were identified and removed. Sequences were then grouped into operational taxonomic units (OTUs) belonging to the same genus. Taxonomic classification was obtained from the RDP (Ribosomal Database Project). Reads classified as the same genus were grouped into phylotypes. A total of 51,645 reads per sample were used.

### 2.5. Oral Cavity Analysis

To evaluate the symmetry between the temporal muscles, facial ridges, and rostro-caudal and latero-lateral movements, the animal was inspected with the oral cavity closed with external palpation of the dental arcade. After collecting the material for oral microbiota analysis, the lesions present were individually evaluated for the presence of peripheral and infundibular caries. Additionally, the occlusal alterations were described. The evaluation was performed with the aid of an adapted oral endoscope (Intraoral Camera, Horse Dental Care, Piracaia, Sao Paulo, Brazil) for horses, using a rigid endoscope 40–70 cm in length, with an optical angle of 50 to 90°. The caries was evaluated according to type, being either peripheral caries or infundibular caries, and according to their severity, ranging from 0 to 4, based on their characteristics, as outlined by Dacre [21] in Table 1 and Table 2.

### 2.6. Statistical Analysis

For microbiota analysis, Mothur software (version 1.31.2) was used. Alpha diversity was indicated by the number of OTUs (number of genera) and the Chao index (richness), as well as the inverse Simpson and Shannon indices (diversity). The comparison between groups was performed using a *t*-test. Beta diversity was assessed using the Jaccard index, which evaluates community composition by identifying which bacteria are present or absent in a community, and the Yue and Clayton index, which assesses community structure by identifying the present bacteria based on their abundance in the community.

Principal coordinate analysis (PCoA) plots, performed in 2 dimensions, were obtained to compare the samples from each group. The Molecular Variance Analysis (AMOVA) was used to compare the composition and structure of the community between the groups. The effect size of the Linear Discriminant Analysis Effect Size (LEfSe) was used to find significant associations between the relative abundances of both groups. In this case, the Kruskal–Wallis test, a non-parametric test, was applied to detect differences between the groups, followed by an unpaired Wilcoxon test, which is a rank sum test.

A Linear Discriminant Analysis (LDA) result greater than 2 was considered significant. For all analyses, a *p*-value < 0.05 was considered significant. The other results obtained were analyzed using R software version 3.6.1, with a descriptive analysis of the variables performed individually, followed by a Shapiro–Wilk test to check for normality.

For the variables that did not meet the statistical assumptions, qualitative variables where the compared data were non-parametric, the Mann–Whitney test (Wilcoxon test) was used. For comparisons involving quantitative (pH) and parametric data, the unpaired Student’s *t*-test was used, considering independent groups (different animals).

## 3. Results

Table 3 describes the bromatological composition of all the feeds used. All animals had been on the same feeding management for at least three years.

Regarding the oral microbiota, the alpha diversity indices are presented in Table 4. These indices characterize the microbial community of the oral cavity. No significant differences were observed between the groups (*p* > 0.05) for the Chao index (which estimates species richness), the inverse Simpson index, or the Shannon index (both of which assess species composition and relative abundance).

**Figure 1:**
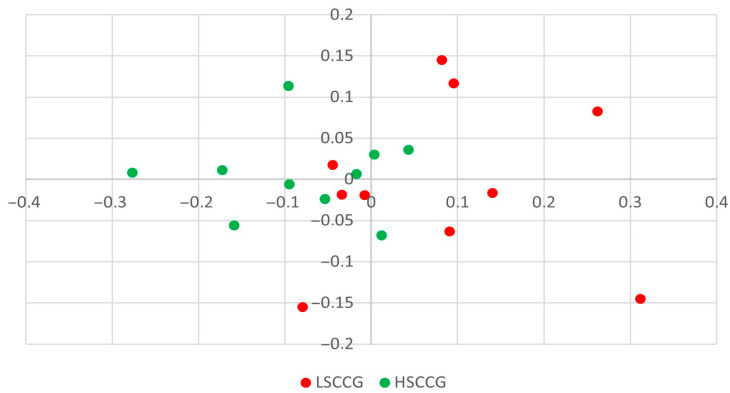
Composition (membership) graph comparing LSCCG (Low Soluble Carbohydrate Content Group) vs. HSCCG (High Soluble Carbohydrate Content Group).

To make observations regarding beta diversity, principal coordinate analysis (PCoA) plots were constructed. In Figure 1, the composition (membership) graph comparing LSCCG vs. HSCCG can be observed, i.e., considering all the bacteria present regardless of their abundance. In this type of graph, the closer the points are to each other, the greater the similarity between the oral microbiota of each horse, and the farther apart they are, the greater the difference. Through this result it was possible to observe that there is no clear pattern of separation between the groups, and for this reason, no significance was observed (*p* = 0.107).

**Figure 2:**
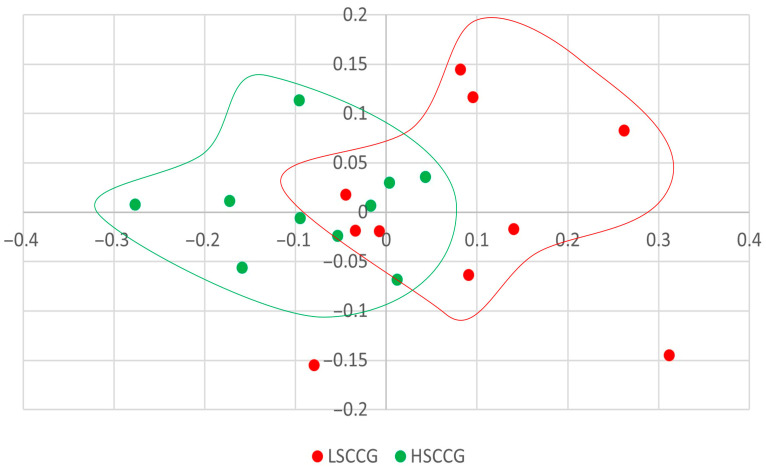
Structure plot comparing LSCCG vs. HSCCG.

Concerning Figure 2, the graph presented is the structure plot, meaning that, in addition to the bacterial composition, the relative abundance of each component of the community is also taken into account; and in this case, it can be observed that there is a clustering of each treatment, indicating a significant difference between LSCCG and HSCCG (*p* = 0.003). This means that, although there may be individual variation in the species of bacteria present in each horse’s oral microbiota, when considering the composition and abundance of each species, a similar profile exists for animals consuming the same type of diet.

**Figure 3:**
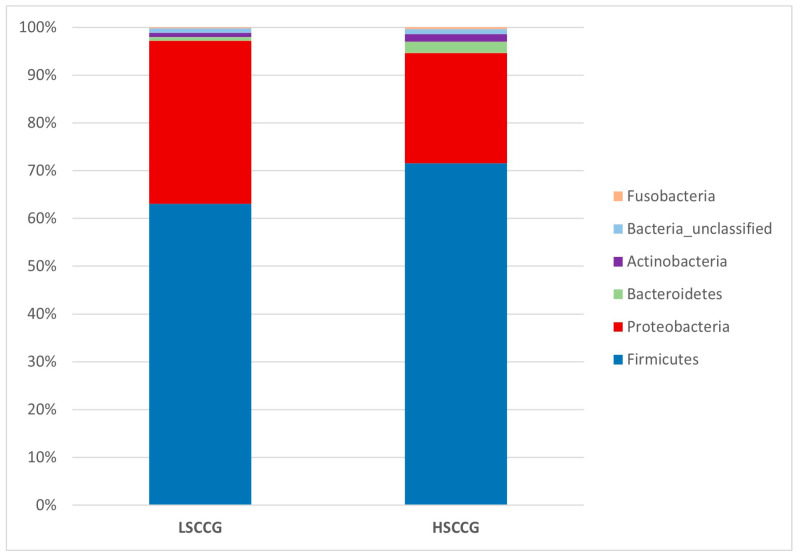
Graph with relative abundance regarding the phyla comparing LSCCG vs. HSCCG.

**Figure 4:**
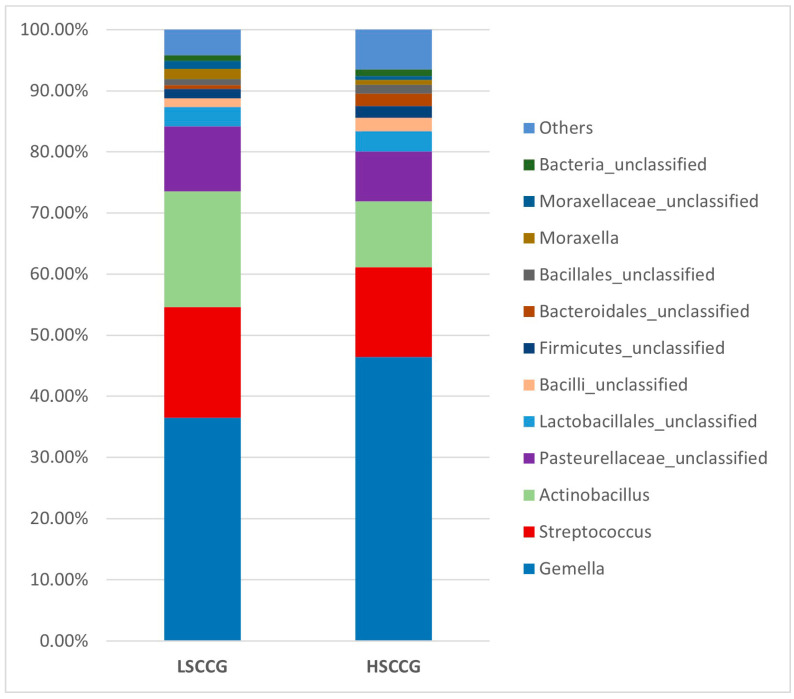
Graph with the relative abundance regarding the genera comparing LSCCG vs. HSCCG.

Subsequently, comparisons were made at the phylum (Figure 3) and genus (Figure 4) levels, considering bacteria with at least 1% relative abundance.

**Figure 5:**
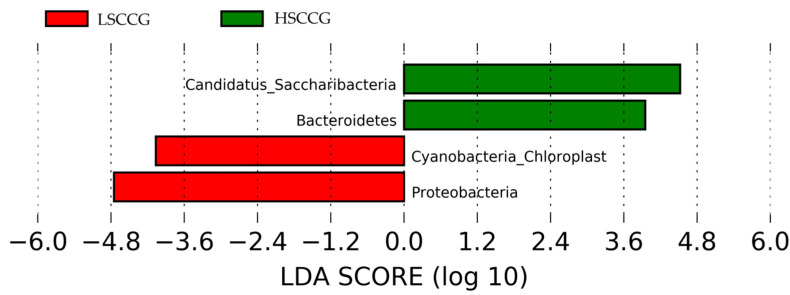
Linear Discriminant Analysis (LDA Score) of phyla that showed significant differences (LDA > 2) comparing LSCCG vs. HSCCG.

**Figure 6:**
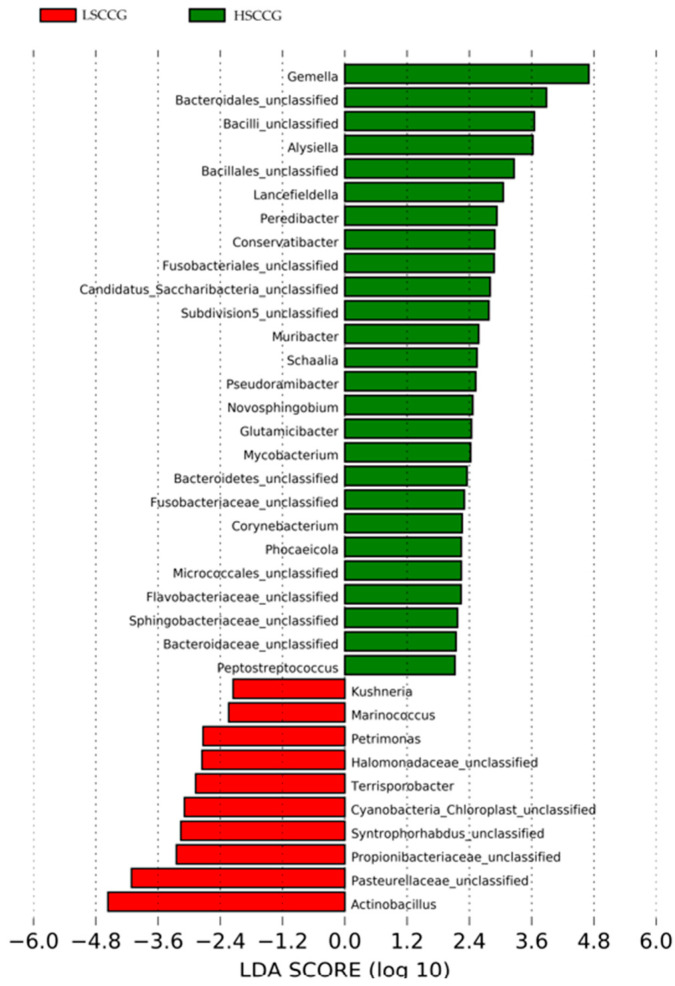
Linear Discriminant Analysis (LDA Score) of genera that showed significant differences (LDA > 2) comparing LSCCG vs. HSCCG.

Additionally, a Linear Discriminant Analysis (LDA Score) was performed in order to demonstrate associations between each bacterial genus and the studied groups. When the LDA value is greater than two for a genus in each group, it implies that this microorganism has a significantly higher relative abundance than in the other group, being considered a potential marker for that type of diet. The same applies to other taxonomic orders such as phylum, class, order, etc. Figure 5 illustrates the phyla *Candidatus Saccharibacteria* and *Bacteroidetes,* which showed a difference in the HSCCG, and *Cyanobacteria_Chloroplast* and *Proteobacteria* for the LSCCG. Regarding the genera, Figure 6 highlights those that were significant (LDA > 2).

**Figure 7:**
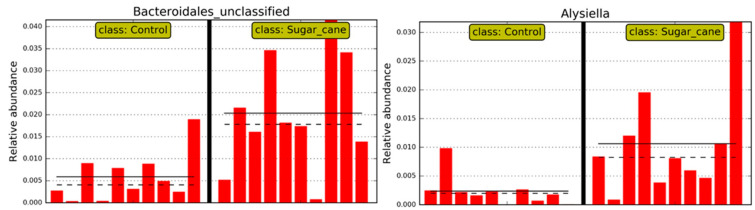
Individual distribution of animals in LSCCG (Control) vs. HSCCG (Sugar_cane).

**Figure 8:**
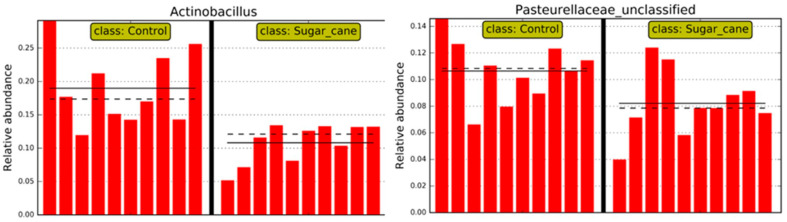
Individual distribution of animals in LSCCG (Control) vs. HSCCG (Sugar_cane).

Thus, regarding the bacterial genera, those that were considered significant in each group LSCCG vs. HSCCG in the Linear Discriminant Analysis (LDA Score) were the ones with an LDA > 2 (Figure 6), and data for each genus were obtained for each animal in the different groups. Therefore, the genera *Bacteroidales* and *Alysiella* were the most discriminative in the HSCCG, and *Actinobacillus* and *Pasteurellaceae_unclassified* in the LSCCG (Figure 7 and Figure 8).

Differences were observed between the groups for the variables “presence of peripheral caries,” “degree of peripheral caries,” and “class of peripheral caries” (Table 5). Regarding the presence of peripheral caries, in the LSCCG, 20% of the animals had peripheral caries in maxillary premolars and molars, 60% in maxillary molars, 10% in mandibular molars, and 10% had no caries; this contrasts with the HSCCG, where 20% had peripheral caries in the maxillary and mandibular molars, and 80% had caries in all quadrants (*p* = 0.001) (Table 5).

Regarding the degree of peripheral caries, which also differed between the evaluated groups, in the LSCCG, 80% of the caries were grade 1, 10% were grade 2, and 10% showed no caries as previously mentioned. In the HSCCG, 10% of the caries were grade 1, 40% were grade 2, 20% were grade 3, and 30% were grade 4 (*p* = 0.01). For the class of peripheral caries, a difference was also observed between the groups. In the LSCCG, 70% were class 1, 20% were class 2, and 10% had no peripheral caries. In the HSCCG, 10% of animals presented peripheral caries of class 1, while 90% presented class 2 (*p* = 0.05).

Concerning the presence of diastemas in the oral cavity, due to the non-parametric and irregular distribution of percentages, a descriptive analysis of the qualitative variables was performed, thus representing the distribution of these alterations in each dental quadrant as percentages, classified according to the Triadan dental notation model (Table 6).

In the meantime, in addition to peripheral caries, this study observed a difference between the groups regarding the presence of open-type diastema, with 10% of the animals in the LSCCG showing this alteration and 70% in the HSCCG. When the comparison was made for valve-type diastema, 40% of the animals in the LSCCG exhibited this alteration, while 50% exhibited it in the HSCCG.

## 4. Discussion

Conceptually, the presence of caries is recognized as a consequence of the interaction between the oral microbiota, dietary habits, dentition, and the oral environment [22]. This study is the first to demonstrate changes in the oral microbiota related to the type of diet received and dental diseases in equines.

The conducted study showed no differences in the total richness of a community based on a sample, that is, in the number of species present in a community, between the equines fed with sugarcane and those fed with *Cynodon* sp. grass, as the Chao index did not show any differences between the groups. Similarly, the inverse Simpson and Shannon indices, which consider the relative abundances of species in a community and provide a deeper analysis of its composition, also showed no differences between the groups, validating the obtained results.

On the other hand, no differences were observed in the Jaccard index, indicating that there was no variation in the community composition, that is, in the bacteria present or absent, but differences were identified for the Yue and Clayton indices (*p* = 0.003), indicating that most of the organisms present in one group were also present in the other but in different proportions. These results demonstrate that there is similarity between the bacterial genera within each studied group. In other words, animals fed with sugarcane had an abundance of bacteria that were similar to each other, and the same occurred for animals fed with *Cynodon* sp. grass.

These data differ from the study conducted by Borkent et al. [8], involving an analysis of the equine peripheral dental microbiota, where no similarity pattern was found in the studied groups for the Yue and Clayton index. Thus, the results found allow for a deeper investigation and observation of these differences in a more specific way and across different taxonomic classes, as performed at the phylum and genus levels. Through the Linear Discriminant Analysis (LDA Score) of phyla, the presence of diet-specific indicator bacteria was observed, such as the phyla *Candidatus Saccharibacteria* and *Bacteroidetes* for high soluble carbohydrate intake and *Cyanobacteria Chloroplast* and *Proteobacteria* for low soluble carbohydrate intake.

Specifically, no difference was found between horses fed sugarcane and those fed *Cynodon* sp. grass (LSCCG vs. HSCCG) regarding infundibular caries occurrence in this study. Research on infundibular caries in horses remains limited, as most of the existing literature focuses on caries in brachydont animals due to similarities with the human dental process [8]. Since infundibular caries are a unique feature of equine dentition, studies are primarily restricted to this species [23,24,25,26,27].

The current understanding suggests that the presence of cemental hypoplasia in the infundibular vascular channels predisposes horses to localized central infundibular caries. When these hypoplastic areas become exposed to the oral cavity due to dental wear, food particles and oral microorganisms can infiltrate these defects, promoting the development of infundibular carious lesions [28].

No differences were observed in the occurrence of infundibular caries between groups, which may be attributed to intrinsic anatomical factors of the maxillary teeth, as well as the lesser influence of diet on this type of lesion compared to peripheral caries. Additionally, the relatively small sample size may have limited statistical power to detect potential differences.

While the findings provide valuable insights, several limitations should be acknowledged. Firstly, the sample size of 10 horses per group is relatively small, which may have reduced the statistical power to detect subtle or moderate differences between groups. As a result, some potentially meaningful effects may have gone undetected. Additionally, the breed of the horses was not specified, and since breed-related anatomical and physiological differences can influence dental morphology and pathology, the lack of breed standardization may limit the applicability of the results to the wider equine population. This, in turn, affects the generalizability of the findings, particularly to breeds or populations with significantly different dental characteristics. Future studies involving larger and more diverse cohorts, including breed-specific analyses, would help to confirm these findings and enhance their external validity.

In research on human oral microbiota, the phylum *Candidatus Saccharibacteria*, formerly known as TM7, was identified based on genomic analysis, which suggested that this phylum of bacteria, characterized by reduced genomes, primarily consumes sugar compounds. Furthermore, members of the TM7 phylum are consistently present in the human oral microbiota, and accumulated evidence associates them with periodontal disease and caries [29]. Similarly, this study observes a significantly higher relative abundance of this same phylum in equines fed a sugarcane-based diet, highlighting the association with high sugar levels in the diet. Moreover, this group of equines exhibited the highest proportions of peripheral caries and diastemas. Therefore, this study represents a pioneering approach, being the first to establish a correlation between the characteristics of the oral microbiota, the type of diet, and dental conditions.

In human medicine, up to 99% of the oral microbiota is represented by the phyla *Firmicutes*, *Actinobacteria*, *Bacteroidetes*, *Proteobacteria*, *Fusobacteria*, *Candidatus Saccharibacteria*, *Spirochaetes*, and *Tenericutes*. Furthermore, the phyla *Actinobacteria* and *Proteobacteria* are predominant in healthy human individuals, while *Bacteroidetes*, *Fusobacteria*, *Candidatus Saccharibacteria*, *Spirochaetes*, and *Synergistes* are more prevalent in individuals with periodontitis [30]. These results are aligned with those of this study, as *Proteobacteria* was also associated with equines fed *Cynodon* sp. grass, and *Candidatus Saccharibacteria* and *Bacteroidetes* were found in equines fed with sugarcane, which also showed a higher presence of peripheral caries and diastemas. In contrast, when comparing the oral microbiota of equines with peripheral caries and healthy animals, Borkent et al. [8] observed a higher abundance of *Actinobacteria* and *Proteobacteria* in the healthy groups, while *Firmicutes* predominated in the group with peripheral caries. In the present study, only *Proteobacteria* showed similar results.

When analyzed at the genus taxonomic level between equines fed with sugarcane and those fed with *Cynodon* sp. grass, it can be observed that when considering LDA > 2, there is a higher quantity of bacteria associated with the diet high in soluble carbohydrates, as observed by Borkent et al. [8]. It was found that more than 35 genera of microorganisms showed differences (LDA > 2) between the groups fed with different levels of soluble carbohydrates. It is likely that there is an increase in synergy between these microorganisms during the oral acidogenic process, contributing to the fermentation of these carbohydrates into tooth-damaging acids, which are exacerbated by the presence of substrates, such as sugars derived from the diet.

Moreover, the analysis of the oral microbiota at the genus taxonomic level is essential to understand the variations in bacterial communities associated with different health conditions. In addition, in the findings of the study by Borkent et al. [8], the genera *Streptococcus* and *Olsenella* were identified as discriminative in the peripheral caries group, a result that differs from the present study, in which the most discriminative genera were *Bacteroidales* and *Alysiella*.

Conversely, Yang et al. [31], when determining the oral microbiota composition of humans with the presence of caries, found *Bacteroidales* (*p* = 0.062) associated with the disease, similar to this study. This is a genus of Gram-negative, rod-shaped, anaerobic bacteria with both motile and non-motile species. They are acidogenic and acidophilic bacteria, with notable species including *Streptococcus mutans*, *Lactobacillus*, acidogenic and aciduric strains of *Neisseria* spp., *Bacteroides* spp., *Actinomyces* spp., and non-mutans *Streptococcus*, which, in combination and in various associations, can cause caries when the ecological conditions of the oral cavity are favorable [32].

Borkent et al. [8] found *Gemella* and *Actinobacillus* as the most discriminative genera in the control group, which aligns with the work of Kennedy et al. [14], who also identified *Gemella* and *Actinobacillus* as the genera most associated with their control group. Gao et al. [33] correlated bacteria found in the subgingival areas of horses considered healthy with bacteria from the genera *Actinobacillus* and *Pasteurellaceae_unclassified*. Similarly, in this study, *Actinobacillus* was associated with equines fed *Cynodon* sp. grass. However, these results differ in relation to *Gemella*, which was associated with equines fed sugarcane in this study. It is possible that this type of diet may have influenced this discrepancy.

The microbial contribution to dental damage goes beyond compositional differences between groups and involves functional traits such as acid production and biofilm formation. Bacteria such as *Bacteroidales* and *Alysiella*, enriched in the HSCCG, are known to possess acidogenic and aciduric characteristics [31,32]. These traits enable them to metabolize fermentable carbohydrates, which are abundant in sugarcane-based diets, into organic acids. This process lowers the pH in the oral cavity and promotes the demineralization of dental tissues [3,6]. Additionally, the formation of stable biofilms allows prolonged acid contact with dental surfaces, exacerbating damage to enamel, dentin, and cementum [22,29]. These mechanisms align with the ecological plaque hypothesis described in human dentistry, where diet-induced dysbiosis promotes the growth of cariogenic bacteria, leading to tissue destruction [30,32]. Therefore, the proliferation of these microbial genera in sugarcane-fed horses likely plays a direct role in the higher incidence and severity of peripheral caries and diastemata observed in this group.

Although only a descriptive analysis of dental changes was conducted, it was detected that equines fed a sugarcane-based diet developed more peripheral caries and diastemas than animals fed with *Cynodon* sp. grass as their primary forage. Similarly, it was observed that both valve-type and open-type diastemas appeared in mandibular tooth quadrants. This finding suggests that, in these teeth, there is a more significant masticatory impact due to the pressure exerted by the maxillary teeth and gravity itself. Additionally, the dental anatomy of this region contributes to a greater susceptibility to these alterations [34].

It is worth noting that the study did not control the sources of concentrate and mineral salt provided to the animals, as selection was made independently of these components. While the primary focus was on forage type, it is possible that variations in these additional dietary elements may have influenced oral pH and microbial composition. Nevertheless, it is known that the type of diet and particularly high amounts of soluble carbohydrates in hay can be associated with an increase in peripheral caries [35], as well as the feeding of fermented forages and concentrates [36]. In this study, it was observed that equines fed with sugarcane had a higher incidence of peripheral caries compared to those fed with *Cynodon* grass. This can be attributed to the dietary profile, which favors fermentation, pH acidification, and the deterioration of dental tissues [7].

## 5. Conclusions

This study demonstrates that long-term dietary intake significantly influences the oral health and microbiota composition of horses. Animals fed a high soluble carbohydrate diet based on sugarcane (HSCCG) exhibited a higher incidence and severity of peripheral caries and diastemata compared to those maintained on a pasture-based diet with *Cynodon* spp. (LSCCG).

Although overall microbial diversity did not differ significantly between groups, distinct microbial profiles were identified, suggesting that specific bacterial taxa may be associated with dietary composition and dental pathology. The bacteria associated with HSCCG were from the genera *Alysiella* and *Bacteroidales*, while in the control group (LSCCG), *Actinobacillus* and *Pasteurellaceae_unclassified* were found.

Through the integrated analysis of microbial community profiles and comprehensive dental examinations, this study offers valuable insights into the complex interplay between dietary composition and the modulation of the equine oral microbiota. The findings underscore the influence of nutritional factors on the structure and diversity of microbial populations within the oral cavity and highlight their potential implications for the onset and progression of dental pathologies, thereby contributing to a deeper understanding of how diet may affect oral health in horses.

## Figures and Tables

**Table 1:** Classification of peripheral caries using the modified Honma system [21].

Degree of Peripheral Caries	Description of Lesions in Dental Tissues
Grade 0	Normal tooth, without visible macroscopic peripheral caries
Grade 1, class 1	Only cementum affected: The lesions appear as spots of superficial or focal erosions
Grade 1, class 2	Only cementum affected: More severe lesions with cementum completely lost in some areas, exposing the underlying enamel (but grossly unaffected)
Grade 2	Lesions affecting the adjacent cementum and enamel
Grade 3	Lesions affecting cementum, enamel, and dentin

**Table 2:** Infundibular caries classification using the modified Honma system [15].

Infundibular Caries Classification	Description of Lesions in Dental Tissues
Grade 0	Normal teeth without visible macroscopic infundibular caries; a small central defect (vascular canal) on the occlusal surface of the infundibula is considered normal
Grade 1	Only cementum affected
Grade 2	Lesions affecting the adjacent cementum and enamel
Grade 3	Lesions affecting cementum, enamel, and dentin
Grade 4	Lesions causing loss of tooth integrity (secondary fractures and/or apical infection)

**Table 3:** Bromatological composition of the feeds used.

Samples	Soluble Carbohydrates	Dry Matter (%)	Ash	Crude Protein	Ether Extract	Starch
Forage						
Sugarcane-based forage (*Saccharum officinarum*)	35.9	95.91	3.04	1.62	0.94	10.49
Napier (Pennisetum purpureum)	6.5	94.94	6.69	3.04	1.71	5.32
Purple Stargrass (Poaceae), (*Cynodon* L. C. Rich)	7.51	95.22	7.41	13.19	1.68	3.67
Tifton 85 (*Cynodon* spp.)	5.55	94.70	7.36	10.83	2.11	1.27
Concentrate						
Homemade ground feed	3.75	86.21	1.78	11.31	3.81	83.53
Homemade meal-type feed	5.29	86.22	1.64	11.08	3.34	82.50
Sustance 12 Farmer Feeds	4.88	90.09	6.90	11.96	3.90	25.33
Proequi 13 Flaked Feed	5.98	88.43	12.43	13.61	3.90	17.31

**Table 4:** Evaluation of alpha diversity indices (*n* = 20).

Item	Experimental Group		*p*-Value
	LSCCG	HSCCG	
Chao1	182.654	213.096	0.1288
Inverse Simpson	4.488	3.958	0.2338
Shannon	1.906	1.960	0.5916

LSCCG: Low Soluble Carbohydrate Group; HSCCG: High Soluble Carbohydrate Group.

**Table 5:** Evaluation of peripheral and infundibular caries in the oral cavity of the evaluated animals (*n* = 20).

Variable Group	Categories	Experimental Group		*p*-Value
		LSCCG	HSCCG	
Presence of peripheral caries	1: Maxillary premolars and molars	2 (20%)	0	0.001
	2: Maxillary molars	6 (60%)	0	
	3: Maxillary and mandibular molars	0	0	
	4: Mandibular molars	1 (10%)	2 (20%)	
	5: All quadrants	0	8 (80%)	
	6: Absent of caries	1 (10%)	0	
Presence of infundibular caries	1: Maxillary premolars	2 (20%)	0	>0.05
	2: Maxillary molars	0	1 (10%)	
	3: All quadrants	1 (10%)	4 (40%)	
	4: Absent of caries	6 (60%)	5 (50%)	
Degree/grade of peripheral caries	1: Degree/grade 1	8 (80%)	1 (10%)	0.01
	2: Degree/grade 2	1 (10%)	4 (40%)	
	3: Degree/grade 3	0	2 (20%)	
	4: Degree/grade 4	0	3 (30%)	
	5: Absent of caries	1 (10%)	0	
Class of peripheral caries	1: Class 1	7 (70%)	1 (10%)	0.05
	2: Class 2	2 (20%)	9 (90%)	
	3: Absent of caries	1 (10%)	0	
Degree/grade of mesial infundibular caries	1: Degree/grade 1	2 (20%)	3 (30%)	>0.05
	2: Degree/grade 2	2 (20%)	0	
	3: Degree/grade 4	0	2 (20%)	
	4: Absent of caries	(60%)	5 (50%)	
Class of mesial infundibular caries	1: Degree/grade 1	1 (10%)	1 (10%)	>0.05
	2: Degree/grade 2	1 (10%)	0	
	3: Degree/grade 3	0	1 (10%)	
	4: Degree/grade 4	0	2 (20%)	
	5: Absent of caries	8 (80%)	6 (60%)	
Class of distal infundibular caries	1: Class 1	1 (10%)	2 (20%)	>0.05
	2: Class 2	0	1 (10%)	
	3: Absent of class and/or caries	9 (90%)	7 (70%)	

**Table 6:** Analysis of the presence of diastema in the valve and in the oral cavity of the evaluated animals (*n* = 20).

Variable Group	Categories	Experimental Group	
		LSCCG	HSCCG
Open diastema	1: Quadrant 1	0	0
	2: Quadrant 2	0	0
	3: Quadrant 3	0	0
	4: Quadrant 4	1 (10%)	1 (10%)
	5: Quadrants 1 and 2	0	1 (10%)
	6: Quadrant 3 and 4	0	4 (40%)
	7: Absent	9 (90%)	3 (30%)
	8: All quadrants	0	1 (10%)
Valve diastema	1: Quadrant 1	1 (10%)	0
	2: Quadrant 2	0	2 (20%)
	3: Quadrant 3	0	0
	4: Quadrant 4	1 (10%)	0
	5: Quadrants 1 and 2	1 (10%)	0
	6: Quadrant 3 and 4	1 (10%)	3 (30%)
	7: Absent	6 (10%)	5 (50%)
	8: All quadrants	0	0

## Data Availability

The original contributions presented in this study are included in the article. Further inquiries can be directed to the corresponding author.

## Abbreviations

The following abbreviations are used in this manuscript:

HSCCG	High Soluble Carbohydrate Group
LSCCG	Low Soluble Carbohydrate Group
PCoA	Principal Coordinate Analysis
LEfSe	Linear Discriminant Analysis Effect Size
LDA	Linear Discriminant Analysis
AMOVA	Molecular Variance Analysis
